# Clinical and Histopathological Characteristics of Granuloma Annulare: Significance of Giant Cells and Systemic Inflammatory Markers

**DOI:** 10.3390/diagnostics16010117

**Published:** 2026-01-01

**Authors:** Zeynep Güngör Hayat, Aziz Hakkı Civriz, Berna Solak

**Affiliations:** 1 Department of Dermatology, Sakarya Training and Research Hospital, 54100 Sakarya, Türkiye; 2 Department of Pathology, Kocaeli University, 41380 Kocaeli, Türkiye; 3 Department of Pathology, Sakarya Training and Research Hospital, 54100 Sakarya, Türkiye; 4 Department of Dermatology, School of Medicine, Sakarya University, 54100 Sakarya, Türkiye

**Keywords:** granuloma annulare, giant cells, basophil-to-lymphocyte ratio, histopathology, prognosis, comorbidities

## Abstract

**Background:** Granuloma Annulare (GA) is a chronic inflammatory dermatosis with diverse presentations, yet comprehensive data integrating histopathology, long-term prognosis, and systemic inflammatory markers remain limited. This study aimed to analyze these clinicopathological characteristics and identify biomarkers for chronicity and recurrence. **Methods:** In this retrospective cohort study, we evaluated 34 patients with histopathologically confirmed GA diagnosed between 2013 and 2023. Demographic data, clinical features, comorbidities, treatment outcomes, and laboratory parameters were analyzed. Histopathological findings were re-evaluated to assess pattern types and the presence of specific markers. Statistical correlations between clinical parameters, inflammatory markers, and histopathological features were assessed. **Results:** The interstitial pattern was the predominant histopathological subtype (64.7%), with universal mucin deposition (100%). A statistically significant positive correlation was observed between lesion duration and the presence of multinucleated giant cells (r = 0.456, *p* = 0.033), suggesting a time-dependent granuloma maturation. Clinically, generalized GA cases demonstrated significantly longer recovery times compared to localized forms. The overall recurrence rate was 23.5%. A lower BLR was significantly associated with disease recurrence (r = −0.539, *p* = 0.021). At least one comorbidity was present in 76.5% of the patients. **Conclusions:** Our findings suggest that giant cell formation serves as a histopathological marker of lesion chronicity, supporting the concept of granuloma remodeling over time. Furthermore, a suppressed BLR may serve as a novel surrogate marker associated with disease recurrence. Given the high burden of comorbidities observed in both localized and generalized forms, GA should be approached as a cutaneous manifestation of underlying systemic dysregulation, suggesting that clinicians should consider routine metabolic screening for these patients.

## 1. Introduction

Granuloma annulare (GA) is a benign, chronic inflammatory dermatosis of unknown etiology, characterized histologically by necrobiotic granulomas [1,2]. Although it is a relatively common condition encountered in dermatology practice, its pathogenesis remains unelucidated. Proposed triggers include trauma, insect bites, viral infections, and ultraviolet radiation, but the precise mechanism of granuloma formation is still debated [2,3].

Clinically, GA presents in various forms with distinct morphological characteristics. The localized form, which represents the most common presentation, typically manifests as asymptomatic, ring-shaped erythematous papules or plaques predominantly located on the dorsal aspects of the hands and feet. In contrast, generalized (or disseminated) GA is characterized by widespread cutaneous involvement and is notoriously recalcitrant to treatment, often requiring systemic therapeutic interventions. While the localized form is often self-limiting, generalized GA can be resistant to therapy and significantly impact the patient’s quality of life. Furthermore, the association of GA with systemic conditions such as diabetes mellitus, thyroid disorders, and dyslipidemia has been a subject of interest, with conflicting results in the literature [1,4]. Among the rarer subtypes, subcutaneous GA (or deep GA) presents as deep, firm nodules without surface changes and is considered an underdiagnosed entity, particularly in pediatric patients [5,6]. The perforating type is a rare variant distinguished by the transepidermal elimination of necrobiotic material. Recent reports have highlighted unique presentations of perforating GA associated with cysts or comedones [7] and rare anatomical involvement such as the subungual region [8].

Comprehensive studies integrating demographic, clinical, laboratory, and detailed histopathological data on GA are scarce. Understanding regional phenotypic variations and histopathological correlations could provide better insights into the disease’s nature. This study aims to retrospectively analyze the clinicopathological characteristics, treatment outcomes, and comorbidity profiles of GA patients diagnosed in a tertiary center and to explore specific histopathological markers that may correlate with clinical parameters.

## 2. Methods

### 2.1. Study Design and Population

In this retrospective cohort study, we analyzed the medical records of patients diagnosed with GA at the Sakarya University Training and Research Hospital Dermatology Outpatient Clinic between November 2013 and December 2023. Inclusion criteria were (1) histopathologically confirmed diagnosis of GA and (2) a minimum follow-up period of two years to adequately assess treatment response and recurrence. Patients with incomplete medical records or uncertain diagnoses were excluded. Ethical approval for this study was obtained from the Sakarya University Faculty of Medicine Health Sciences Ethics Committee (Approval No: E-43012747-050.04-409971-73, Date: 14 October 2024).

### 2.2. Data Collection and Histopathological Evaluation

Demographic data (age, gender), clinical features (lesion site, number, duration), laboratory results (complete blood count, biochemistry, autoimmune markers), and treatment modalities were recorded. Baseline laboratory parameters were retrieved from medical records corresponding to the time of initial diagnosis, prior to the administration of treatment. Systemic inflammatory markers, including the Neutrophil-to-Lymphocyte Ratio (NLR), Monocyte-to-Lymphocyte Ratio (MLR), Basophil-to-Lymphocyte Ratio (BLR), and C-reactive protein-to-Albumin Ratio, were calculated from the complete blood count and biochemistry results.

Operational Definitions: In this study, generalized GA was operationally defined as the presence of 10 or more lesions, or widespread involvement affecting at least two distinct anatomical regions (such as the trunk and extremities). This classification aligns with criteria frequently cited in comparable retrospective cohorts, such as Dabski et al. [9]. Furthermore, recurrence was defined as the reappearance of visible lesions at either the original site or new sites, following a documented period of complete clinical clearance lasting at least 3 months.

Histopathological Review: All archival Hematoxylin and Eosin (H&E) (Harris Hematoxylin Solution, Sigma-Aldrich, Merck KGaA, Darmstadt, Germany) stained slides were re-evaluated by an experienced dermatopathologist using a light microscope (Olympus BX51, Olympus Corporation, Tokyo, Japan). To ensure objectivity, the review was performed blinded to the patients’ clinical recurrence status. Mucin deposition was confirmed using Alcian Blue (pH 2.5) staining (Artisan Alcian Blue/PAS Stain Kit, Agilent Technologies, Santa Clara, CA, USA) in all cases. Subsequently, it was graded semi-quantitatively (Absent; Mild: focal stringy basophilia; Severe: diffuse pools of mucin). Similarly, necrobiosis intensity was graded as mild (focal collagen degeneration) or severe (large necrobiotic areas).

### 2.3. Statistical Analysis

Statistical analysis was performed using the Python programming language version 3.x (Python Software Foundation, Wilmington, DE, USA). The normality of data distribution was assessed using the Kolmogorov–Smirnov test and Q-Q plots. Continuous variables were compared using Student’s *t*-test or Mann–Whitney U test, depending on distribution. Categorical variables were compared using the Chi-square test or Fisher’s Exact test. Correlations between variables (e.g., lesion duration vs. histopathological features) were evaluated using Point-Biserial and Spearman correlation analyses. The association between BLR and recurrence was evaluated as an exploratory analysis using point-biserial correlation. A *p*-value of <0.05 was considered statistically significant.

## 3. Results

### 3.1. Demographic and Clinical Characteristics

Initially, 41 patients with histopathologically confirmed granuloma annulare were identified. However, patients with incomplete follow-up data or a follow-up duration of less than two years were excluded from the study. Consequently, a total of 34 patients were included in the final analysis. The majority were female (73.5%, *n* = 25), with a mean age of 62.2 ± 13.9 years. The localized form was observed in 67.6% (*n* = 23) of patients, while 32.4% (*n* = 11) presented with generalized GA. The anatomical distribution of lesions is detailed in Table 1. The most common site was the upper extremities (38.2%), followed by multiple site involvement (38.2%).

### 3.2. Histopathological Findings

**Figure 1:**
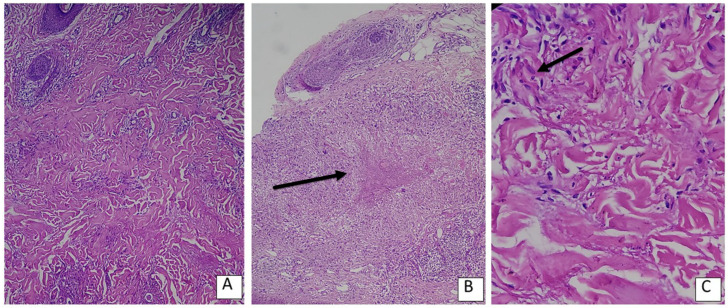
(**A**) The interstitial pattern, which was the most prevalent subtype in our study, characterized by a ‘busy dermis’ appearance with lymphohistiocytic infiltration insinuating between collagen bundles without overt granuloma formation (H&E, ×100). (**B**) The classic palisading granuloma pattern, showing a central zone of necrobiosis (black arrow) surrounded by radially arranged lymphohistiocytes (H&E, ×100). (**C**) High-power view demonstrating a multinucleated giant cell (black arrow) within the inflammatory infiltrate, accompanied by degenerated collagen fibers (necrobiosis) and nuclear dust (karyorrhexis) (H&E, ×400).

The interstitial pattern was the most prevalent histopathological subtype (64.7%), followed by the palisading granuloma pattern (23.5%). Lymphohistiocytic infiltration was observed in 70.6% of cases. Notably, a statistically significant moderate positive correlation was found between the presence of giant cells and the duration of lesions (r = 0.456, *p* = 0.033). Mild-to-severe mucin deposition was present in all cases (Table 2). Representative histopathological examples demonstrating the predominant interstitial pattern, the classic palisading granuloma with multinucleated giant cells, and necrobiotic areas containing nuclear dust are shown in Figure 1.

### 3.3. Comorbidities and Laboratory Findings

The most prevalent comorbidities were diabetes mellitus and hypertension, both observed in 15 patients (44.1%). Coronary artery disease and thyroid disorders (including goiter, hypothyroidism, and nodules) were each identified in 3 patients (8.8%). At least one comorbidity was present in 76.5% (*n* = 26) of the patients. Multiple (≥2) concomitant systemic diseases were observed in 19 patients (55.9%) (Table 3). Regarding autoimmune markers, Antinuclear Antibody (ANA)positivity was detected in 36.0% (9/25), and Anti-thyroid Peroxidase (Anti-TPO)positivity was found in 44.4% (4/9) of tested patients. No statistically significant difference was found in the frequency of comorbidities between generalized and localized patients (*p* = 0.388).

A detailed comparison of demographic, clinical, and laboratory characteristics stratified by disease extent (localized vs. generalized) is presented in Table 4.

### 3.4. Treatment and Prognosis

Topical corticosteroids were the most common treatment modality (88.2%). Systemic treatments were required for 17.6% of patients (Table 5). The overall recurrence rate was 23.5%. Recurrence was observed in 5 of the 23 patients (21.7%) with localized GA and in 3 of the 11 patients (27.3%) with generalized GA. No statistically significant difference was found regarding recurrence rates (*p* > 0.999). A significant positive correlation was observed between generalized involvement and recovery time (r = 0.685, *p* < 0.001). The median recovery time was significantly longer in generalized GA cases (6 months) compared to localized cases (2 months).

**Figure 2:**
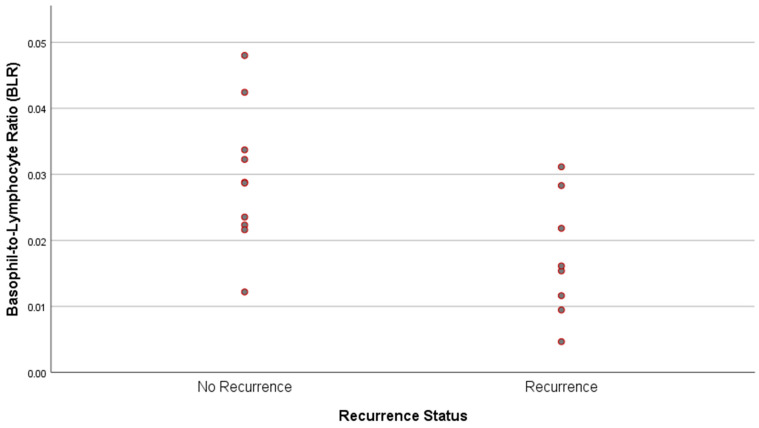
Scatter plot illustrating the distribution of Basophil-to-Lymphocyte Ratio (BLR) values according to recurrence status.

Correlation analysis revealed a statistically significant moderate negative correlation between the recurrence status and the basophil-to-lymphocyte ratio (BLR) (r = −0.539, *p* = 0.021) (Figure 2). In contrast, no statistically significant correlations were observed between recurrence and other evaluated parameters.

## 4. Discussion

The salient findings of this analysis provide new insights into the clinicopathological and prognostic spectrum of Granuloma Annulare (GA) in our cohort. First, our study reinforces the diagnostic dominance of the interstitial histopathological pattern, identifying mucin deposition as a universal feature (100%) that is indispensable for diagnosis in subtle cases. Second, we report a novel histopathological correlation between lesion duration and the presence of giant cells, suggesting a chronological “maturation” of the granuloma potentially driven by elastophagocytosis. Third, from a prognostic perspective, we identified that a suppressed Basophil-to-Lymphocyte Ratio (BLR) is significantly associated with disease recurrence, likely reflecting a heightened Th1-polarized inflammatory state, while generalized forms demonstrated a significantly longer recovery time compared to localized cases. Finally, our data underscores a substantial burden of metabolic comorbidities, particularly diabetes and hypertension, which appear to drive a sustained systemic inflammatory response regardless of the extent of cutaneous involvement.

Although the literature regarding GA has been increasing in recent years, it remains limited. A comprehensive, large-scale study conducted by Barbieri et al. [10] in the United States reported the annual incidence of GA as 0.04% and the prevalence as 0.06%. Our demographic data aligns with these global trends, particularly regarding female predominance. We found a female-to-male ratio of approximately 3:1, consistent with the findings of the aforementioned study [10] as well as other recent international cohorts [11].

However, the mean age in our cohort (62.2 years) was notably higher than the mean age of 48.3 years reported by Chatterjee et al. [12] in India and exceeds the peak incidence age of 40–50 years typically reported in Western literature [4,9,10]. This discrepancy may be attributed to the tertiary care setting of our study, where younger patients with asymptomatic, localized lesions might be underrepresented due to delayed medical consultation. Additionally, given the high prevalence of comorbidities in our cohort, the elevated mean age suggests that GA in this population may be more closely associated with age-related metabolic dysregulation rather than idiopathic triggers [13].

The localized form was the predominant phenotype in our cohort (67.6%), aligning closely with data reported by Cyr [4] (75%). Clinically, we observed a significantly longer recovery time in the generalized form compared to localized cases. This finding corroborates recent cohorts documenting the recalcitrant nature of disseminated GA, which often requires systemic therapies and exhibits a protracted course compared to the self-limiting localized subtypes [2,6,9,14,15].

In our evaluation of systemic inflammatory markers, including CRP, NLR, MLR, and the CRP-to-albumin ratio, we observed no statistically significant differences between the generalized and localized GA groups. To the best of our knowledge, the English-language literature lacks comprehensive studies specifically comparing these hematological inflammatory indices between GA phenotypes. While the immunopathogenesis of GA has been primarily characterized at the cutaneous level, particularly by T-cell activation, interleukin-2 expression, and predominance of Th1-related pathways with additional involvement of Th2 and JAK–STAT signaling, our findings suggest that this pronounced local immune activation does not necessarily translate into a systemic inflammatory response capable of distinguishing clinical phenotypes [16,17]. The absence of significant variation in systemic markers suggests that the extent of cutaneous involvement (localized vs. generalized) may not correlate with the magnitude of systemic inflammation. Instead, as supported by the elevated HbA1c levels in our cohort and recent large-scale association studies, the systemic aspect of GA may be driven more by chronic metabolic dysregulation rather than an acute inflammatory surge [13].

An intriguing finding of our study was the statistically significant negative correlation between the Basophil-to-Lymphocyte Ratio (BLR) and recurrence rates. Patients with lower BLR values were significantly more prone to disease recurrence. Since GA is characterized as a Th1-mediated delayed-type hypersensitivity reaction, this finding may be interpreted through two synergistic mechanisms [16,17]. First, the systemic predominance of Th1 cytokines (e.g., IFN-gamma) may actively suppress the Th2-associated basophilic axis in the bone marrow. Second, the decrease in circulating basophils might reflect their active recruitment and consumption at the cutaneous inflammatory site (peripheral sequestration). In either scenario, a suppressed BLR appears to serve as a surrogate marker for a heightened and persistent pro-inflammatory state, identifying a disease phenotype that is immunologically active and predisposed to relapse despite initial clinical improvement.

In the histopathological spectrum of GA, our study corroborates the shifting consensus from the ‘classic’ palisading pattern to the interstitial variant. We observed the interstitial pattern in 64.7% of cases, which aligns closely with the study by Ronen et al. [18] (71%), though it is higher than the 44% reported by Chatterjee et al. [12]. Interestingly, a recent study by Bagci et al. observed that the interstitial pattern was the predominant subtype in malignancy-associated GA, contrasting with the necrobiotic pattern seen in classic cases, which further highlights the potential systemic significance of this histological variant [19]. The predominance of the interstitial pattern is clinically significant as it often presents a diagnostic challenge due to its subtle histological features (‘busy dermis’). In this context, the presence of mucin becomes a critical diagnostic discriminator. While Chatterjee et al. [12] reported dermal mucin in 80% and other studies up to 93% of cases [1,9], we detected mild-to-severe mucin deposition in all cases (100%). This finding underscores that mucin evaluation should be an indispensable part of the diagnostic workup, particularly in subtle interstitial cases.

Regarding the cellular infiltrate, we identified a lymphohistiocytic predominance in 70.6% of patients, consistent with the chronic inflammatory nature of the disease. While typical GA presents with a mixed infiltrate, rare variants such as ‘pseudolymphomatous GA’ characterized by dense lymphoid aggregates have also been described, further illustrating the broad histological spectrum and immunological complexity of the disease [20]. However, the most novel and salient finding of our study is the statistically significant positive correlation between lesion duration and the presence of giant cells. While histiocytes are the hallmark of GA, our data suggests that the formation of multinucleated giant cells is a time-dependent phenomenon. While histiocytes are the hallmark of GA, our data suggests that the formation of multinucleated giant cells is a time-dependent phenomenon. This ‘maturation’ of the granuloma aligns with recent spatial transcriptomics and mechanistic studies demonstrating dynamic macrophage polarization and cytokine cascades (e.g., JAK-STAT pathways) in granuloma formation [21,22]. This evolution can be explained by two synergistic mechanisms: first, the persistent release of Th1-associated cytokines (TNF-alpha and IFN-gamma) promoting macrophage fusion over time [16,17]; and second, the progressive elastolytic activity in the dermis, where giant cells are increasingly recruited to engulf degenerated elastic fibers (elastophagocytosis) with increasing chronicity, a process sharing histopathological features with annular elastolytic giant cell granuloma [21].

Diabetes mellitus and hypertension were the most prevalent comorbidities in our cohort, each affecting 44.1% of patients. The prevalence of diabetes in our study is notably higher than the 21.1% reported in the large-scale population-based study by Barbieri et al. [13], a discrepancy likely attributable to the advanced mean age of our study population compared to previously reported cohorts. Regarding hypertension, while direct comparisons are limited as cardiovascular comorbidities were not the primary outcome in the aforementioned analysis, our findings reinforce the concept of a substantial metabolic burden in this patient group [23,24]. Crucially, we observed no statistically significant difference in comorbidity rates between localized and generalized cases. This lack of distinction suggests that the extent of cutaneous involvement does not necessarily reflect the severity of systemic associations. Therefore, we suggest that clinicians consider screening for metabolic syndrome and thyroid autoimmunity in GA patients, as our findings imply that even localized lesions may signal systemic risk. Although the high Anti-TPO positivity rate (44.4%) observed in our tested cohort might be partially influenced by selection bias, the sheer magnitude of this association cannot be overlooked and justifies routine evaluation.

The management of granuloma annulare, particularly the generalized form, presents a significant therapeutic challenge [2,3]. Our study empirically validates this clinical observation, demonstrating a statistically significant delay in time to recovery for generalized cases (median 6 months) compared to localized forms (median 2 months). Furthermore, we observed an overall recurrence rate of 23.5%, with recurrence occurring in 27.3% of generalized cases and 21.7% of localized cases. Although the difference in recurrence rates did not reach statistical significance (*p* > 0.999), these findings confirm the chronic and relapsing nature of the disease irrespective of the clinical subtype, as previously described in the literature [4,9].

Our study has certain limitations inherent to its retrospective design. First, being a single-center study conducted in a tertiary care setting, our cohort may reflect a more recalcitrant disease spectrum compared to the general population, which could account for the higher mean age and comorbidity burden observed. Second, the lack of a healthy control group prevents us from establishing definitive relative risks for systemic markers. Third, due to the retrospective nature of data collection, comprehensive laboratory screening (such as thyroid autoantibodies) was not uniform across all patients, which may introduce a selection bias regarding specific comorbidity rates. Finally, although the histopathological re-evaluation was performed in a blinded manner to minimize bias, it was conducted by a single experienced dermatopathologist. Despite these constraints, this study represents a unique contribution to the existing literature. To the best of our knowledge, it is the first comprehensive evaluation to simultaneously analyze histopathological patterns, long-term clinical outcomes (recovery time and recurrence), and systemic inflammatory markers within the same patient cohort.

## 5. Conclusions

In conclusion, our study highlights the interstitial pattern as the predominant histopathological variant of Granuloma Annulare in our cohort, with mucin deposition serving as a valuable diagnostic discriminator. The correlation observed between giant cell formation and lesion duration suggests a chronological evolution of the granuloma, supporting the concept of ‘granulomatous maturation.’ Additionally, we identify a lower Basophil-to-Lymphocyte Ratio (BLR) as a potential marker associated with recurrence. The prolonged recovery observed in generalized cases underscores the recalcitrant nature of this phenotype. Finally, the substantial burden of metabolic and autoimmune comorbidities across both localized and generalized forms suggests that GA may be associated with underlying systemic dysregulation. Consequently, we suggest that clinicians consider routine screening for metabolic syndrome and thyroid autoimmunity in GA patients to ensure comprehensive patient care.

## Figures and Tables

**Table 1:** Anatomical distribution of lesions (*n* = 34).

Anatomical Site	*n* (%)
Upper extremities	13 (38.2)
Hand	9 (26.5)
Hands and arms	1 (2.9)
Arms	3 (8.8)
Lower extremities	4 (11.8)
Foot	2 (5.9)
Leg	2 (5.9)
Trunk and Neck	4 (11.8)
Trunk	2 (5.9)
Trunk and neck	1 (2.9)
Neck	1 (2.9)
Multiple regions	13 (38.2)
Hands, arms, and neck	3 (8.8)
Hands, arms, and trunk	3 (8.8)
Arms and legs	3 (8.8)
Arms and trunk	2 (5.9)
Generalized	2 (5.9)

**Table 2:** Comparison of histopathological findings according to lesion extent.

	Localized GA (*n* = 23)	Generalized GA (*n* = 11)	*p*-Value
Histopathological Subtype			
Interstitial	16 (69.6%)	6 (54.5%)	
Palisading granuloma	6 (26.1%)	2 (18.2%)	0.264
Mixed	0	1 (9.1%)	
Sarcoidal	0	1 (9.1%)	
Subcutaneous	1 (4.3%)	1 (9.1%)	
Mucin Deposition			
Absent	0	0	
Mild	10 (43.5%)	8 (72.7%)	0.218
Severe	13 (56.5%)	3 (27.3%)	
Cell Type in Infiltrate			
Lymphohistiocytic	15 (65.2%)	9 (81.8%)	0.437
Mixed	8 (34.8%)	2 (18.2%)	
Necrobiosis Intensity			
Absent	5 (21.7%)	2 (18.2%)	
Mild	8 (34.8%)	5 (45.5%)	0.905
Severe	10 (43.5%)	4 (36.4%)	
Additional Findings			
Nuclear Dust	19 (82.6%)	7 (63.6%)	0.388
Palisading	14 (60.9%)	8 (72.7%)	0.705
Giant Cells	12 (52.2%)	7 (63.6%)	0.715

**Table 3:** Summary of comorbidities and autoimmune markers in the study cohort (*n* = 34).

Parameter	*n* (%)
Comorbidities	
Diabetes Mellitus	15 (44.1%)
Hypertension	15 (44.1%)
Coronary Artery Disease	3 (8.8%)
Thyroid Disorders	3 (8.8%)
Presence of ≥1 Comorbidity	26 (76.5%)
Multiple Comorbidities (≥2)	19 (55.9%)
Autoimmune Markers	
ANA Positivity ^a^	9 (36.0%)
Anti-TPO Positivity ^b^	4 (44.4%)

^a^ Calculated based on 25 patients tested. ^b^ Calculated based on 9 patients tested.

**Table 4:** Comparison of demographic, clinical, and laboratory characteristics stratified by disease extent.

	Localized (*n* = 23)	Generalized (*n* = 11)	*p*-Value
Sex			
Female	16 (69.6%)	9 (81.8%)	0.682
Male	7 (30.4%)	2 (18.2%)	
Age (years)	62.22 ± 15.56	62.00 ± 10.32	0.962
Any comorbidity	19 (82.6%)	7 (63.6%)	0.388
Disease duration at Diagnosis (months)	7.50 (1.00–60.00)	7.00 (3.00–36.00)	0.580
Time to recovery (months)	2.00 (1.00–12.00)	6.00 (6.00–36.00)	<0.001
Recurrence	5 (21.7%)	3 (27.3%)	>0.999
WBC count (10^3^/µL)	7.00 (4.06–9.76)	7.49 (6.17–10.39)	0.311
Neutrophil count (10^3^/µL)	4.15 (1.85–6.15)	4.06 (3.58–5.22)	0.927
Lymphocyte count (10^3^/µL)	2.04 (0.86–4.58)	2.44 (1.53–4.30)	0.101
Monocyte count (10^3^/µL)	0.52 (0.27–0.93)	0.56 (0.37–0.76)	0.645
Eosinophil count (10^3^/µL)	0.13 (0.04–2.67)	0.13 (0.07–2.20)	0.941
Basophil count (10^3^/µL)	0.05 (0.01–0.17)	0.06 (0.02–0.12)	0.517
C-reactive protein (CRP) (mg/L)	3.85 (2.70–16.30)	5.05 (2.80–16.00)	0.810
Thyroid stimulating hormone (TSH) (mIU/L)	1.89 (0.36–8.37)	1.36 (0.52–3.49)	0.597
HbA1c (%)	6.30 (4.80–15.30)	7.00 (5.10–10.50)	>0.999
Ferritin (µg/L)	32.60 (1.73–238.00)	57.95 (16.20–78.00)	0.340
Albumin (g/dL)	43.40 (33.40–46.80)	42.00 (41.40–47.70)	0.721
Neutrophil-to-lymphocyte ratio	1.98 (0.57–4.94)	1.75 (1.21–2.69)	0.274
Monocyte-to-lymphocyte ratio	0.22 (0.07–1.03)	0.23 (0.17–0.27)	0.971
Basophil-to-lymphocyte ratio	0.02 (0.00–0.09)	0.02 (0.00–0.06)	0.971
CRP-to-albumin ratio	0.09 (0.06–0.44)	0.21 (0.06–0.39)	0.905

**Table 5:** Treatment modalities administered to patients (*n* = 34).

	*n* (%)
Topical Therapies	
Corticosteroids	30 (88.2)
Corticosteroids + Tacrolimus	2 (5.9)
Intralesional corticosteroids	1 (2.9)
Systemic Therapies	
Corticosteroids	1 (2.6)
Corticosteroids + Hydroxychloroquine	1 (2.6)
Corticosteroids + Methotrexate	1 (2.6)
Hydroxychloroquine	2 (5.1)
Hydroxychloroquine + Methotrexate	1 (2.6)

Note: Five patients (14.7%) received both systemic and localized therapies concomitantly.

## Data Availability

The data presented in this study are available on request from the corresponding author. The data are not publicly available due to privacy and ethical restrictions regarding patient confidentiality.
